# A phenological shift in the time of recruitment of the shipworm, *Teredo navalis* L., mirrors marine climate change

**DOI:** 10.1002/ece3.2126

**Published:** 2016-05-10

**Authors:** Christin Appelqvist, Jonathan N Havenhand

**Affiliations:** ^1^ Sven Lovén Centre for Marine Infrastructure– TjärnöUniversity of GothenburgSE‐452 96 StrömstadSweden; ^2^ Department of Marine Sciences – TjärnöUniversity of GothenburgSE‐452 96 StrömstadSweden

**Keywords:** Marine borer, phenology, sea surface temperature, teredinidae, warming

## Abstract

For many species, seasonal changes in key environmental variables such as food availability, light, and temperature drive the timing (“phenology”) of major life‐history events. Extensive evidence from terrestrial, freshwater, and marine habitats shows that global warming is changing the timings of many biological events; however, few of these studies have investigated the effects of climate change on the phenology of larval recruitment in marine invertebrates. Here, we studied temperature‐related phenological shifts in the breeding season of the shipworm *Teredo navalis* (Mollusca, Bivalvia). We compared data for the recruitment period of *T. navalis* along the Swedish west coast during 2004–2006 with similar data from 1971–1973, and related differences in recruitment timing to changes in sea surface temperature over the same period. We found no significant shift in the timing of onset of recruitment over this ~30‐year time span, but the end of recruitment was an average of 26 days later in recent years, leading to significantly longer recruitment periods. These changes correlated strongly with increased sea surface temperatures and coincided with published thermal tolerances for reproduction in *T. navalis*. Our findings are broadly comparable with other reports of phenological shifts in marine species, and suggest that warmer sea surface temperatures are increasing the likelihood of successful subannual reproduction and intensifying recruitment of *T. navalis* in this region.

## Introduction

There is substantial evidence that climate warming is driving changes in the Earth′s biological systems (Parmesan 2006; Thackeray et al. 2010; Donnelly et al. 2011; Wernberg et al. 2011; Stocker et al. 2014). Shifts have been observed in species distributions (Philippart et al. 2011), species abundance and population dynamics (Richardson 2008; Mieszkowska et al. 2009), and the timing of seasonal behaviors and events (McCarty 2001; Morgan et al. 2013), among others. The effect of climate warming on phenology – the timing of recurrent biological events with respect to the environment – has mostly been studied in terrestrial ecosystems (e.g., Khanduri et al. 2008; Diamond et al. 2011; Vitasse et al. 2014; Buentgen et al. 2015; Navarro‐Cano et al. 2015; Way and Montgomery 2015). Although less is known about the phenological responses of marine species to rapid warming of the oceans, the last decade has seen a rapid increase in our understanding of this issue (Sydeman and Bograd 2009; Lett et al. 2010; Donnelly et al. 2011; Poloczanska et al. 2013). For example, phenological shifts have been reported for plankton (Calbet et al. 2014; Villarino et al. 2015), benthos (Philippart et al. 2003, 2014; Moore et al. 2011; Richards 2012), and fish (Perry et al. 2005; Neidetcher et al. 2014; Asch 2015). These have in turn raised concerns about the synchrony of interactions and possible mismatches between different trophic levels (Beaugrand et al. 2003; Donnelly et al. 2011; Atkinson et al. 2015).

Following this trend, climate‐related range shifts have been reported for several species of shipworm (Mollusca: Teredinidae) in Europe (Borges et al. 2014). More recent work, however, has found no evidence for range extension in at least one of these species, *Teredo navalis*, in Sweden (Appelqvist et al. 2015a) – a result that is also supported by climate envelope modeling (Appelqvist et al. 2015b). Interestingly, that modeling also suggested that over the last few decades the breeding season has extended later into the summer/autumn and that this is likely to continue (Appelqvist et al. 2015b). To date, there are no published long‐term data on shipworm recruitment in the region against which these observations and projections can be evaluated.

Shipworms play an important role in degrading wood in the ocean, which they burrow into and consume, in the process causing substantial damage to man‐made marine wooden structures (Turner 1966; Nair and Saraswathy 1971; Paalvast 2014). The common shipworm, *Teredo navalis* (L.), is globally distributed and eurythermal. Adults are typically active at water temperatures of 5–30**°**C, but can survive down to 0**°**C (Roch 1932; Nair and Saraswathy 1971). *Teredo navalis* is a protandrous hermaphrodite and “spermcaster”: males release sperm freely into the water column, whereas females retain eggs within the epibranchial cavity, into which sperm are drawn and fertilization occurs. Embryos and larvae are brooded within the epibranchial cavity to “D‐stage” veliger larvae (Culliney 1975) and released into the water column at temperatures ≥16**°**C (Loosanoff and Davis 1963). Typically, after 15–20 days of feeding and growth in the plankton, larvae have acquired competence to settle onto wood substrata (Grave 1928; Loosanoff and Davis 1963; Culliney 1975). Growth of shipworms is highly temperature dependent, especially in temperate seas (Nair and Saraswathy 1971). In Scandinavian waters, *T. navalis* shows highest growth rates at temperatures ≥15**°**C (Roch 1932; Kristensen 1979). Generation times in *T. navalis* are relatively short (40–50 day, Grave 1928, 1942), and given sufficiently warm temperatures and adequate food, sexual maturity can be attained within just a few weeks of settlement and metamorphosis (Grave 1928), leading to multiple generations within a breeding season (Hoppe 2002).

In Swedish waters, *T. navalis* is close to its northern range margin in the eastern Atlantic (Turner 1966; Borges et al. 2014). Sea surface temperatures in this area have increased significantly the last decades (Philippart et al. 2011; and refs. therein), raising the possibility that the breeding season may have changed, perhaps facilitating subannual reproduction. We investigated the phenology of recruitment of *T. navalis* in western Sweden and compared our results to historical data from an identical survey conducted 35 years earlier (Norman 1976). Further, we assessed whether changes in the phenology of recruitment of *T. navalis* over this period were related to sea surface warming in the region.

## Materials and Methods

### Sampling and study site

**Figure 1:**
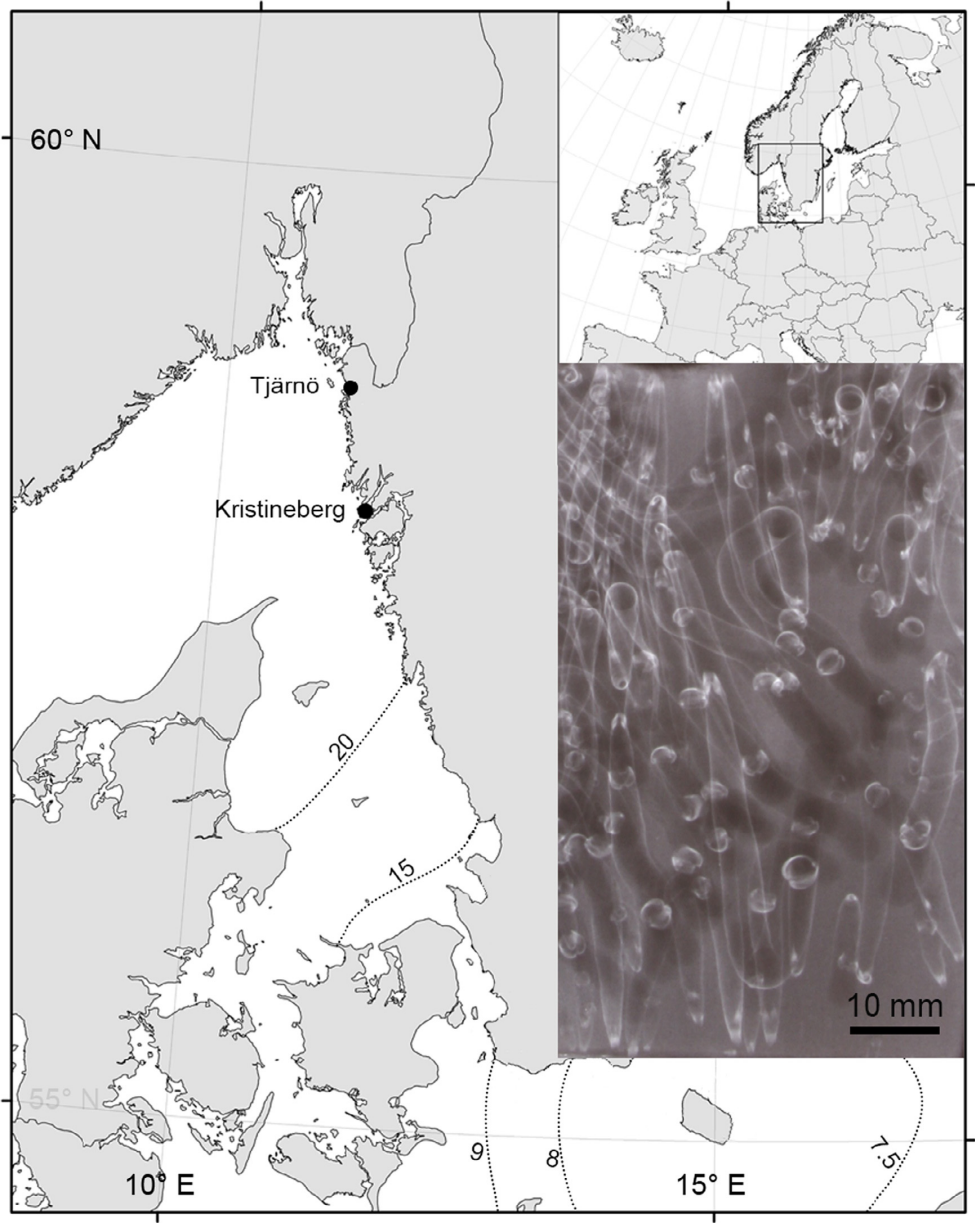
Location of the study sites. Dotted lines indicate isohalines, numbers indicate mean annual surface salinity. Inset: radiograph of part of a shipworm‐infested panel.

Recruitment of shipworm larvae was assessed over three successive years: 2004, 2005, and 2006 at the Sven Lovén Centre, Kristineberg, western Sweden (N 58**°**14′57″, E 11**°**20′50″, Fig. 1). Methods were designed to follow as closely as possible those used by Norman (1976), the only differences being that we freeze‐stored exposed panels prior to X‐ray analysis, and used a different type of X‐ray apparatus.

Recruitment was measured onto untreated pine (*Pinus sylvestris*) panels, 20 × 75 × 200 mm, placed at 0.5, 1.5, and 2.5 m depth (the maximum water depth at the sampling site was 6 m). The onset of larval recruitment was assessed by submerging multiple sets of panels (one panel at each depth) in early June. After 14 days, and every 14 days thereafter, one set of these panels was then retrieved, all macroscopic fouling on the surface of the panels was removed, and panels were stored at −20°C for later analysis. The end of the larval recruitment period was determined by submerging equivalent sets of panels every 14 days, starting in early June. Panels were left in the water until collection in November, at which point all panels were processed as outlined above.

### Determining shipworm abundance on panels

Whole panels were X‐radiographed using an Andrex BV 155 portable X‐ray machine (30 kV/3 mA). Shipworm recruitment intensity was defined as the number of visible individual (or pairs of) shells ≥2 mm on the radiograph, expressed per unit area (overall panel area = 0.015 m^2^, Fig. 1, Norman 1976). Thus, “recruitment” was defined as the time at which newly settled individuals were first observed, *sensu* Keough and Downes (1982).

### Time of recruitment

The beginning and end of the recruitment period were defined in two ways. First, we estimated the onset (and end) of intense recruitment using statistical fits of logistic growth models to cumulative recruitment data. Onset of intense recruitment was estimated from the intercept of the maximal recruitment rate (logistic “growth” rate) with the date (*x*) axis (or its equivalent for the end of intense recruitment). We also used these same models to obtain statistical estimates of the rates of recruitment at the beginning and end of the recruitment periods, respectively. Relevant parameters of best‐fit logistic models, and their 95% CI's, were obtained using the package *grofit* within the R statistical environment (Kahm et al. 2010; R Development Core Team, 2010). These statistically derived parameters are broadly equivalent to the “arrival intensity” measures of Denny et al. (2014). Secondly, we recorded the first (and last) day on which we observed new recruits on our panels. The day of first observation is equivalent to “first arrival date” (FAD, Tryjanowski and Sparks 2001). We termed the corresponding last arrival date “LAD”.

Possible shifts in time of recruitment between the 1970s and 2000s were determined by comparing the metrics outlined above for our own data to equivalent values that we calculated for data extracted from Norman (1976). All data were assessed for homogeneity of variances using Levene's test prior to analysis (in no cases were the results of this test significant) and compared using *t*‐tests.

### Temperature

Daily sea surface temperature (SST) data were obtained for the study site on the Swedish west coast. Data from 1971 to 1973 were taken from logbooks at the Sven Lovén Centre – Kristineberg. Equivalent data for 2004–2006 were not available, and we therefore used temperature data from the Sven Lovén Centre – Tjärnö, 75 km to the north (Lovén Centre, 2015, Fig. 1). Tests of available temperature data from the two sites during the 2000s showed that these were strongly correlated (*r* ^2^ *= *0.95, *n* = 60, *P *<* *0.0001), and similar (*T°*_*K'berg*_ = 0.911.*T°*_*Tjärnö*_ + 1.35, pairwise comparisons of temperatures; May–July, *t*_1.54_ = 0.150, *P *=* *0.882, August–October, *t*_1.48_ = 0.035, *P *=* *0.972). Changes in SST between the 1970s and 2000s were analyzed using *t*‐tests for “early” (May–July) and “late” (August–October) summer months for all 3 years in each decade, corresponding to the onset and end of recruitment, respectively.

Relationships between SST and the time of recruitment were examined with linear regression. For each year (*n* = 6), the onset of recruitment (intense, and FAD) was regressed against increasing SST (first day of the year with 3‐day mean temperature ≥16**°**C). The end of recruitment (intense, and LAD) was regressed against declining SST (the last 3 days of the year with mean temperature ≥16**°**C). The 16**°**C criterion was based on the minimum reported temperature for release of larvae in *T. navalis* (Loosanoff and Davis 1963).

## Results

### Time of recruitment

**Figure 2:**
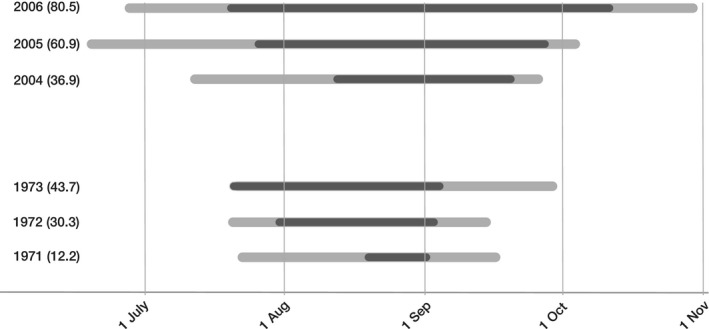
Recruitment of *Teredo navalis* at Kristineberg, Sweden, in the 2000s and 1970s (1970s data from Norman 1976). Black bars show duration of intense recruitment based on statistical fits of recruitment intensity over time (see text). Gray bars indicate the total period over which recruitment was observed (first–last observed recruitment). Numbers within brackets are duration (d) of intense recruitment.

From 2004 to 2006, the onset, duration, and end of shipworm recruitment varied markedly among years. Recruitment was observed as early as mid‐June (in 2005) and as late as the end of October (in 2006; Fig. 2). Averaged across all 3 years, intense recruitment began on 29th July ± 6.7 days and ended on 27th Sept ± 6.2 days. Corresponding dates for first (FAD) and last (LAD) observed recruits were 30th June ± 6.4 days and 7th October ± 9.9 days, respectively (Fig. 2, Table 1, all data means ± SEM).

**Table 1:** Day of the year (DOY) of onset, end, and rate (recruits.day^−1^) of intense recruitment of *Teredo navalis*. All data are obtained from statistical fits of logistic growth models (see text for details)

Year	Onset of recruitment				End of recruitment			
	DOY	95% CI	Rate	95% CI	DOY	95% CI	Rate	95% CI
1971	230.1	7.52	4.72	1.94	242.2	5.69	15.8	7.37
1972	210.9	10.2	4.95	2.43	244.9	3.46	6.98	1.05
1973	201.3	6.38	12.9	6.37	245.0	3.05	21.2	11.6
2004	223.3	5.72	13.0	5.23	260.2	4.60	9.82	2.39
2005	206.5	9.98	3.98	1.14	267.3	15.8	5.32	2.58
2006	200.6	3.82	17.4	7.32	281.1	4.62	8.05	2.08

Comparing these data with those for 1971–1973, it is clear that the period of intense recruitment during the 2000s was longer (mean periods 59.3 days vs. 29.7 days, respectively; black bars, Fig. 2). This was not due to earlier onset: although the day of the year (DOY) on which intense recruitment began was 4 days earlier in the 2000s this was not significantly different (*t*_1.4_ = 0.363, *P *=* *0.735). The end of intense recruitment was, however, significantly later in the year in the 2000s (by 25.9 days, *t*_1.4_ = 4.183, *P *=* *0.014).

Similar decadal differences were observed for the period between FAD and LAD (gray bars, Fig. 2). In comparison with the 1970s, FAD was on average 21.0 days earlier (*t*_1.4_ = 3.253, *P *=* *0.031), and LAD was 19.3 days later in the 2000s (although the latter was not statistically significant, *t*_1.4_ = 1.781, *P *=* *0.150).

**Figure 3:**
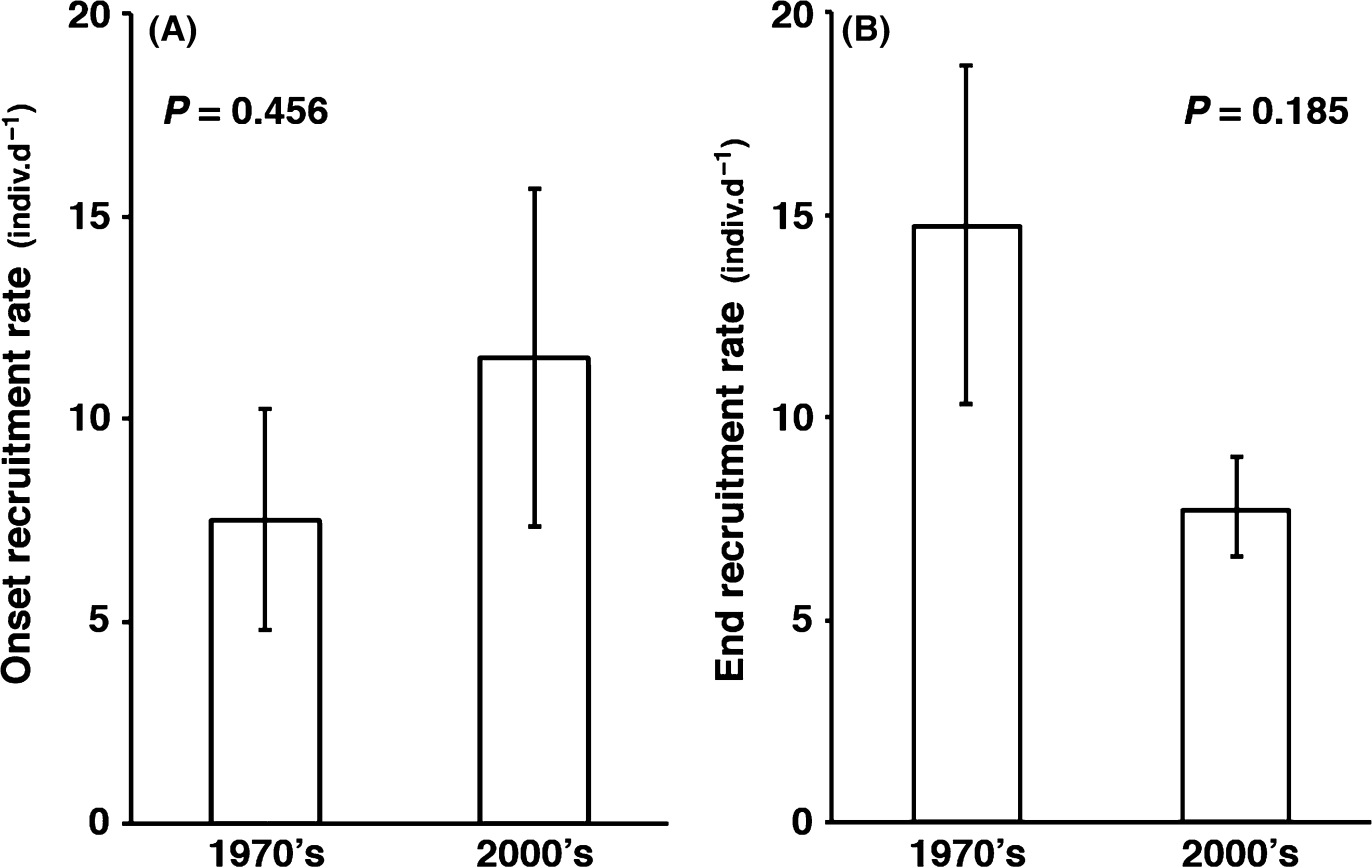
Rate of recruitment during onset (A), and end (B), of intense recruitment period for 1971–1973 and 2004–2006. Rate determined from statistical fits of logistic growth rates (see text for details). *P*‐values indicate significance of *t*‐test of differences between decades, error bars are ± S.E.

Rates of intense recruitment tended to be higher during onset, and lower during end of recruitment, in the 2000s (Fig. 3, Table 1) but these trends were not statistically significant (*t*_1.4_ = 0.825, *P *=* *0.456; *t*_1.4_ = 1.598, *P *=* *0.185, respectively).

### Temperature

Summer SST increased significantly between 1971 and 2006. Mean SST was 1.08**°**C higher in early summer (May–July) and 2.19°C higher in late summer (August–October) in 2004–2006 compared to 1971–1973 (*t*_1.355_ = 2.73, *P *=* *0.007; *t*_1.342_ = 6.32, *P *≤* *0.000; for early and late summer, respectively; see also Fig. S1).

**Figure 4:**
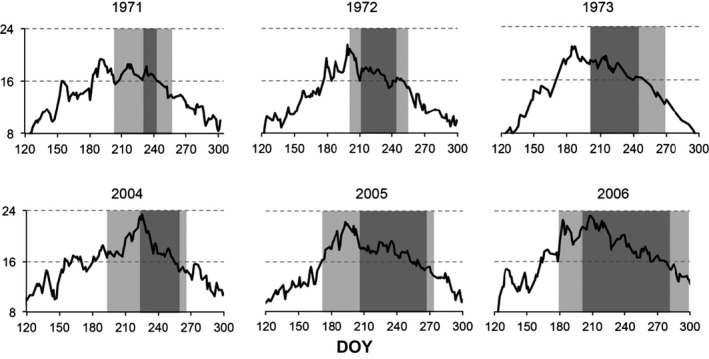
Daily sea surface temperature May–November 1971–1973 (day of year, DOY), from Kristineberg, and 2004–2006 from Tjärnö (see text for details). Dark gray bars show intense recruitment period, light gray shading shows time between first and last arrival of recruits. Data from 1970s taken from Norman (1976).

**Figure 5:**
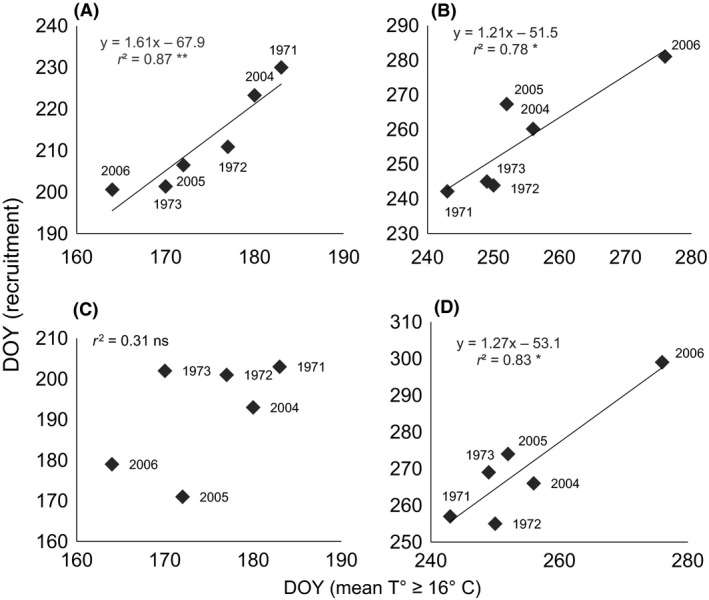
Timing of recruitment and sea surface temperatures (SST) ≥16°C for 1971–1973 and 2004–2006. (A) onset of intense recruitment, (B) end of intense recruitment, (C) first arrival day (FAD), (D) last arrival day (LAD). Data for 1971–1973 are from Norman (1976). **P <* *0.01, *P <* *0.05, ns *P *>* *0.05.

The timing of recruitment showed strong relationships with temperature (Figs. 4 and 5). Across all years, the onset of intense recruitment was significantly correlated with the first DOY on which mean SST ≥16°C (*P = *0.007; Fig. 5a). Similarly, the end of intense recruitment varied significantly with the last DOY on which mean SST ≥16**°**C (*P = *0.02, Fig. 5b). The last day on which recruits were observed (LAD) was also significantly correlated with the last day on which mean SST ≥16°C (*P = *0.01, Fig. 5d); however, there was no equivalent relationship for FAD and the first date on which mean SST ≥16°C (*P = *0.248, Fig. 5c).

## Discussion

Our results show clearly that the length of the recruitment period of shipworms in western Sweden has increased significantly over the last 35 years and that this has occurred in concert with significant summer warming of the sea surface. This shift is apparent in both of the phenological metrics we used: the statistically determined onset/end of intense larval recruitment (a standardized measure of phenological activity, Denny et al. 2014); and the more traditional, but less robust, first arrival day and last arrival day (FAD and LAD, Fig. 3, Sparks et al. 2001). Statistical metrics of phenology also showed a trend for recruits to arrive more rapidly (greater numbers of new recruits per day), and tail‐off more slowly, in recent years in comparison with the 1970s (Fig. 3). This latter result indicates that overall recruitment intensity may have been higher in recent years; however, our data do not permit evaluation of this issue. By designing our assays after Norman (1976), we maximized comparability between studies (decades); however, this also precluded quantification of peak recruitment intensity during the summer because our recruitment assays were almost certainly saturated. Nonetheless, these findings collectively indicate that over the last 35 years, recruitment of *Teredo navalis* continues longer into late summer and may have become more intense.

The first day of the year on which recruits were observed (“first arrival date”, FAD) is a convenient, and commonly used, phenological metric (Tryjanowski and Sparks 2001; Diamond et al. 2011; Benard 2015). FAD is, however, susceptible to strong interannual variation in weather, dispersal patterns, and population size, and consequently several authors have criticized its utility for quantifying the arrival time of a population (Sparks et al. 2001; Tryjanowski and Sparks 2001). Alternate metrics such as mean arrival date across a number of locations, or statistical estimates of arrival date from multiple observations of arrival intensity over time, have been suggested to better describe phenologies (Hüppop 2003; Denny et al. 2014). The logistic models we used to estimate the onset, and end, of intense recruitment (Table 1) provide this latter category of metric. As these are more robust than single observations of FAD and LAD (last arrival day), we focus the remainder of our discussion on statistical estimates of onset and end of intense recruitment.

Our finding that the onset of intense recruitment did not differ significantly between decades, suggests that the phenology of processes leading up to recruitment – such as gonad maturation, spawning, and larval development – has not changed substantively over this period. Intense recruitment in our samples occurred marginally earlier in the season (−4 days, *P *=* *0.735), and at significantly warmer temperatures (+1.08°C, *P *=* *0.007) than in the 1970s. This phenological shift is equivalent to 1.21 days, decade^−1^. A recent meta‐analysis of over 1700 observations of climate effects on temperate marine species found a statistically significant 4.4 ± 0.7 days.decade^−1^ warming‐related shift in spring phenologies (Poloczanska et al. 2013). While these values do not coincide, they are clearly close. Importantly, Poloczanska et al. (2013) emphasized the importance of additional driving factors such as nutrient availability, algal blooms, solar irradiance, and thermal stratification in determining phenologies in the ocean. Consequently, given our relatively small dataset – and a correspondingly large error in estimating phenology shift – it is perhaps reasonable to conclude that the rate of warming‐driven change in the phenology of intense recruitment that we observed in *T. navalis* is similar to that of spring/summer phenologies in many other marine species (Poloczanska et al. 2013).

In contrast to patterns for the onset of recruitment, the end of the recruitment period was substantially, and significantly, later in recent years (by 25.9 days, Fig. 2). This extension was also associated with significantly warmer sea surface temperatures in late summer (+2.19°C, *P *≤* *0.000). Similar climate‐related shifts in reproductive phenology have been reported for many other marine species (e.g., shrimps, Richards 2012; zooplankton, Beaugrand et al. 2009), and once again, this appears to be a highly generalized pattern (Poloczanska et al. 2013). For shipworms, the observed extension of recruitment later into autumn may have been caused by prolonged reproduction of established adults, and/or by rapid maturation and reproduction of early recruits within a summer. The latter possibility is supported by the observations of Grave (1942) and Imai et al. (1950) that newly settled *T. navalis* can become sexually mature within ~45 days. From 1971 to 1973, the mean duration of intense larval recruitment was 29.7 days (Table 1), which would probably have been too short a time for even the very earliest settlers to grow to maturity and reproduce. In 2004–06, however, this period was twice as long (59.3 days, Table 1), providing more than enough time for early recruits to grow, reproduce, and for their larvae to recruit successfully. More rapid juvenile growth and maturation under the warmer summers of recent years may have accelerated this process. Our observation that the rate of decline of recruitment in the autumn was lower in recent years (Fig. 3b) may also be a reflection of the small numbers of recruits that would be produced by newly mature adults of small body size.

In a broader context, the influence of temperature on reproduction, recruitment, and growth of marine organisms is well established (O'Connor et al. 2007). Our findings of such correlations at the onset and end of recruitment (Fig. 5) were based on reports that adult *T. navalis* release larvae at temperatures above 16°C (Loosanoff and Davis 1963). For the onset of recruitment, the delay between the date on which 3‐day mean SST ≥16°C and the onset of intense recruitment was approximately 38 days (Fig. 5a). Using Culliney's (1975) estimate of a 15–20 day larval period prior to settlement, this would imply that postlarvae grew for ~3 weeks before they could be detected on our radiographs. This estimate is supported by our observations that first recruitment was observed in panels that had been exposed to seawater for ≤28 days (FAD data). Timing of the end of recruitment was strongly correlated with the last day on which 3‐day mean SST ≥16°C (Fig. 5b), such that the last day of recruitment occurred ~5 days after the average temperature fell below 16°C. Applying the estimates of larval development and postsettlement growth times outlined above, this would imply that shipworms ceased releasing larvae ~30 days before the average temperature fell below 16°C, that is, at a time when SST was approximately 18°C (Fig. 4). As far as we are aware this is the first estimate of the temperature at which shipworms cease reproduction in the field.

The finding that *T. navalis* in western Sweden are now recruiting later into the autumn is consistent with recent climate envelope modeling. Appelqvist et al. (2015b) suggest that local climate change has increased the risk of recruitment intensity, but not the risk of eastward geographic spread, of shipworms in the western Baltic (*cf* Borges et al. 2014). The population studied here is close to the northern range limit for this species (Borges et al. 2014), and consequently, it would be of value to survey the extent – if any – of poleward extension of this limit. Certainly, our results indicate that even in cooler climates further to the north, summer temperatures should be sufficient to permit successful reproduction and recruitment. This warrants further study.

In summary, we show that over a 35‐year period from the early 1970s to the mid‐2000s, there were substantial changes to the phenology of recruitment in the shipworm, *Teredo navalis,* in western Sweden. These changes were characterized primarily by extension of the end of the recruitment period into the autumn. These changes correlated strongly with concomitant increases in sea surface temperatures and reflect other reports of climate‐related changes in phenology of marine species. This prolongation of the recruitment period will increase the likelihood of successful recruitment of *T. navalis* into areas at the margins of its current range.

## Conflict of Interest

None declared.

## Supporting information


**Figure S1**. Sea Surface Temperature (SST) in western Sweden for July–September 1970–2010.

## References

[ece32126-bib-0001] Appelqvist, C. , J. N. Havenhand , and G. B. Toth . 2015a Distribution and abundance of teredinid recruits along the Swedish coast–are shipworms invading the Baltic Sea? J. Mar. Biol. Assoc. U.K. 95:783–790.

[ece32126-bib-0002] Appelqvist, C. , Z. K. Al‐Hamdani , P. R. Jonsson , and J. N. Havenhand . 2015b Climate envelope modeling and dispersal simulations show little risk of range extension of the shipworm, *Teredo navalis* (L.), in the Baltic Sea. PLoS One 10. doi:10.1371/journal.pone.0119217. 10.1371/journal.pone.0119217 PMC4359003 25768305

[ece32126-bib-0003] Asch, R. G. 2015 Climate change and decadal shifts in the phenology of larval fishes in the California Current ecosystem. Proc. Natl Acad. Sci. USA 112:E4065–E4074. 26159416 10.1073/pnas.1421946112 PMC4522805

[ece32126-bib-0004] Atkinson, A. , R. A. Harmer , C. E. Widdicombe , A. J. McEvoy , T. J. Smyth , D. G. Cummings . 2015 Questioning the role of phenology shifts and trophic mismatching in a planktonic food web. Prog. Oceanogr. 137:498–512.

[ece32126-bib-0005] Beaugrand, G. , K. M. Brander , J. A. Lindley , S. Souissi , and P. C. Reid . 2003 Plankton effect on cod recruitment in the North Sea. Nature 426:661–664. 14668864 10.1038/nature02164

[ece32126-bib-0006] Beaugrand, G. , C. Luczak , and M. Edwards . 2009 Rapid biogeographical plankton shifts in the North Atlantic Ocean. Glob. Change Biol. 15:1790–1803.

[ece32126-bib-0007] Benard, M. F. 2015 Warmer winters reduce frog fecundity and shift breeding phenology, which consequently alters larval development and metamorphic timing. Glob. Change Biol. 21:1058–1065. 10.1111/gcb.12720 25263760

[ece32126-bib-0008] Borges, L. M. S. , L. M. Merckelbach , I. Sampaio , and S. M. Cragg . 2014 Diversity, environmental requirements, and biogeography of bivalve wood‐borers (Teredinidae) in European coastal waters. Front. Zool. 11(1):13. 24520913 10.1186/1742-9994-11-13 PMC3925441

[ece32126-bib-0009] Buentgen, U. , L. Hellmann , W. Tegel , S. Normand , I. Myers‐Smith , A. V. Kirdyanov . 2015 Temperature‐induced recruitment pulses of Arctic dwarf shrub communities. J. Ecol. 103:489–501.

[ece32126-bib-0010] Calbet, A. , A. F. Sazhin , J. C. Nejstgaard , S. A. Berger , Z. S. Tait , L. Olmos , et al. 2014 Future climate scenarios for a coastal productive planktonic food web resulting in microplankton phenology changes and decreased trophic transfer efficiency. PLoS One 9:doi:10.1371/journal.pone.0094388. 10.1371/journal.pone.0094388 PMC3983207 24721992

[ece32126-bib-0011] Culliney, J. 1975 Comparative larval development of the shipworms *Bankia gouldi* and *Teredo navalis* . Mar. Biol. 29:245–251.

[ece32126-bib-0012] Denny, E. G. , K. L. Gerst , A. J. Miller‐Rushing , G. L. Tierney , T. M. Crimmins , C. A. Enquist , et al. 2014 Standardized phenology monitoring methods to track plant and animal activity for science and resource management applications. Int. J. Biometeorol. 58:591–601. 24458770 10.1007/s00484-014-0789-5 PMC4023011

[ece32126-bib-0013] Diamond, S. E. , A. M. Frame , R. A. Martin , and L. B. Buckley . 2011 Species’ traits predict phenological responses to climate change in butterflies. Ecology 92:1005–1012. 21661561 10.1890/10-1594.1

[ece32126-bib-0014] Donnelly, A. , A. Caffarra , and B. F. O'Neill . 2011 A review of climate‐driven mismatches between interdependent phenophases in terrestrial and aquatic ecosystems. Int. J. Biometeorol. 55:805–817. 21509461 10.1007/s00484-011-0426-5

[ece32126-bib-0015] Grave, B. H. 1928 Natural history of shipworm, *Teredo navalis*, at Woods Hole, Massachusetts. Biol. Bull. 55:260–282.

[ece32126-bib-0016] Grave, B. H. 1942 The sexual cycle of the shipworm, *Teredo navalis* . Biol. Bull. 82:438–445.

[ece32126-bib-0017] Hoppe, K.N . 2002 *Teredo navalis* – the cryptogenic shipworm Pp. 116–119 *in* Leppäkoski E., Gollasch S., eds. Invasive aquatic species in Europe: distribution, impacts and management. Kluwer, Dordrecht, Netherlands.

[ece32126-bib-0018] Hüppop, O. 2003 North Atlantic Oscillation and timing of spring migration in birds. Proc. Royal Soc. London B: Biol. Sci. 270:233–240. 10.1098/rspb.2002.2236 PMC1691241 12614571

[ece32126-bib-0019] Imai, T. , M. Hatanaka , and R. Sato . 1950 Breeding of marine timber‐borer *Teredo navalis* L., in tanks and its use for anti‐boring test. Tohoku J. Agric. Res. 1:199–208.

[ece32126-bib-0020] Kahm, M. , G. Hasenbrink , H. Lichtenberg‐Frate , J. Ludwig , and M. Kschischo . 2010 grofit: fitting Biological Growth Curves with R. J. Stat. Softw. 33:1–21.20808728

[ece32126-bib-0021] Keough, M. J. , and B. J. Downes . 1982 Recruitment of marine invertebrates: the role of active larval choices and early mortality. Oecologia 54:348–352. 10.1007/BF00380003 28309958

[ece32126-bib-0022] Khanduri, V. , C. Sharma , and S. Singh . 2008 The effects of climate change on plant phenology. Environmentalist 28:143–147.

[ece32126-bib-0023] Kristensen, E. S. 1979 Observations on growth and life‐cycle of the shipworm *Teredo navalis* L (Bivalvia, Mollusca) in the Isefjord, Denmark. Ophelia 18:235–242.

[ece32126-bib-0024] Lett, C. , S.‐D. Ayata , M. Huret , and J.‐O. Irisson . 2010 Biophysical modelling to investigate the effects of climate change on marine population dispersal and connectivity. Prog. Oceanogr. 87:106–113.

[ece32126-bib-0025] Loosanoff, V. L. , and H. C. Davis . 1963 Rearing of bivalve mollusks. Adv. Mar. Biol. 1:1–130.

[ece32126-bib-0026] Lovén Centre 2015 Sven Lovén centrum för marina vetenskaper ‐ Vatten och väderdata. http://www.weather.loven.gu.se, accessed 15‐01‐2015.

[ece32126-bib-0027] McCarty, J. P. 2001 Ecological consequences of recent climate change. Conserv. Biol. 15:320–331.

[ece32126-bib-0028] Mieszkowska, N. , M. J. Genner , S. J. Hawkins , and D. W. Sims . 2009 Effects of climate change and commercial fishing on Atlantic Cod *Gadus morhua* . Adv. Mar. Biol. 56:213–273. 19895976 10.1016/S0065-2881(09)56003-8

[ece32126-bib-0029] Moore, P. J. , R. C. Thompson , and S. J. Hawkins . 2011 Phenological changes in intertidal con‐specific gastropods in response to climate warming. Glob. Change Biol. 17:709–719.

[ece32126-bib-0030] Morgan, E. , R. M. O'Riordan , and S. C. Culloty . 2013 Climate change impacts on potential recruitment in an ecosystem engineer. Ecol. Evol. 3:581–594. 23532482 10.1002/ece3.419 PMC3605848

[ece32126-bib-0031] Nair, N.B. , and M. Saraswathy , 1971 The Biology of Wood‐Boring Teredinid Molluscs Pp. 335–509 *in* Frederick S.R., Maurice Y., eds. Advances in marine biology. Academic Press, Academic Press, London.

[ece32126-bib-0032] Navarro‐Cano, J. A. , B. Karlsson , D. Posledovich , T. Toftegaard , C. Wiklund , J. Ehrlen , et al. 2015 Climate change, phenology, and butterfly host plant utilization. Ambio 44:S78–S88. 25576283 10.1007/s13280-014-0602-z PMC4289000

[ece32126-bib-0033] Neidetcher, S. K. , T. P. Hurst , L. Ciannelli , and E. A. Logerwell . 2014 Spawning phenology and geography of Aleutian Islands and eastern Bering Sea Pacific cod (*Gadus macrocephalus*). Deep‐Sea Res. Part II‐Top. Stud. Oceanogr. 109:204–214.

[ece32126-bib-0034] Norman, E. 1976 The time of settlement on the swedish west coast of the wood‐boring molluscs *Teredo navalis*,* Psiloteredo megotara* and *Xylophaga dorsalis* . Mater. Organismen 3:531–542.

[ece32126-bib-0035] O'Connor, M. I. , J. F. Bruno , S. D. Gaines , B. S. Halpern , S. E. Lester , B. P. Kinlan , et al. 2007 Temperature control of larval dispersal and the implications for marine ecology, evolution, and conservation. Proc. Natl Acad. Sci. 104:1266–1271. 17213327 10.1073/pnas.0603422104 PMC1764863

[ece32126-bib-0036] Paalvast, P. 2014 Ecological studies in a man‐made estuarine environment, the port of Rotterdam. Nijmegen University, Thesis.

[ece32126-bib-0037] Parmesan, C. 2006 Ecological and evolutionary responses to recent climate change. Annu. Rev. Ecol. Evol. Syst. 37:637–669.

[ece32126-bib-0038] Perry, A. L. , P. J. Low , J. R. Ellis , and J. D. Reynolds . 2005 Climate change and distribution shifts in marine fishes. Science 308:1912–1915. 15890845 10.1126/science.1111322

[ece32126-bib-0039] Philippart, C. J. , H. M. Van Aken , J. J. Beukema , O. G. Bos , G. C. Cadée , R. Dekker . 2003 Climate‐related changes in recruitment of the bivalve *Macoma balthica* . Limnol. Oceanogr. 48:2171–2185.

[ece32126-bib-0040] Philippart, C. J. , R. Anadón , R. Danovaro , J. W. Dippner , K. F. Drinkwater , S. J. Hawkins , et al. 2011 Impacts of climate change on European marine ecosystems: observations, expectations and indicators. J. Exp. Mar. Biol. Ecol. 400:52–69.

[ece32126-bib-0041] Philippart, C. J. , J. D. Van Bleijswijk , J. C. Kromkamp , A. F. Zuur , and P. M. Herman . 2014 Reproductive phenology of coastal marine bivalves in a seasonal environment. J. Plankton Res. 36:1512–1527.

[ece32126-bib-0042] Poloczanska, E. S. , C. J. Brown , W. J. Sydeman , W. Kiessling , D. S. Schoeman , P. J. Moore , et al. 2013 Global imprint of climate change on marine life. Nat. Clim. Chang. 3:919–925.

[ece32126-bib-0043] R Development Core Team 2010 R: a language and environment for statistical computing. R Foundation for Statistical Computing, Vienna, Austria pp Page, ISBN 3–900051–07–0, URL: http://www. R‐project. org.

[ece32126-bib-0044] Richards, R. 2012 Phenological shifts in hatch timing of northern shrimp Pandalus borealis. Mar. Ecol. Prog. Ser. 456:149–158.

[ece32126-bib-0045] Richardson, A. J. 2008 In hot water: zooplankton and climate change. ICES J. Mar. Sci.: J. du Conseil 65:279–295.

[ece32126-bib-0046] Roch, F . 1932 Einige Beobachtungen zur Ökologie und Physiologie von Teredo navalis L, Almqvist & Wiksell, Uppsala, Sweden.

[ece32126-bib-0047] Sparks, T. H. , D. R. Roberts , and H. Q. P. Crick . 2001 What is the value of first arrival dates of spring migrants in phenology? Avian Ecol. Behav. 7:75–85.

[ece32126-bib-0048] Stocker, T. , D. Qin , G.‐K. Plattner , et al. 2014 Climate change 2013: the physical science basis, Cambridge University Press, Cambridge, UK, and New York.

[ece32126-bib-0049] Sydeman, W. J. , and S. J. Bograd . 2009 Marine ecosystems, climate and phenology: introduction. Mar. Ecol. Prog. Ser. 393:185–188.

[ece32126-bib-0050] Thackeray, S. J. , T. H. Sparks , M. Frederiksen , et al. 2010 Trophic level asynchrony in rates of phenological change for marine, freshwater and terrestrial environments. Glob. Change Biol. 16:3304–3313.

[ece32126-bib-0051] Tryjanowski, P. , and T. Sparks . 2001 Is the detection of the first arrival date of migrating birds influenced by population size? A case study of the red‐backed shrike *Lanius collurio* . Int. J. Biometeorol. 45:217–219. 11769324 10.1007/s00484-001-0112-0

[ece32126-bib-0052] Turner, R. D. 1966 A survey and illustrated catalogue of the Teredinidae. Museum of Comparative Zoology, Harvard University, Cambridge, MA.

[ece32126-bib-0053] Villarino, E. , G. Chust , P. Licandro , M. Butenschoen , L. Ibaibarriaga , A. Larranaga , et al. 2015 Modelling the future biogeography of North Atlantic zooplankton communities in response to climate change. Mar. Ecol. Prog. Ser. 531:121–142.

[ece32126-bib-0054] Vitasse, Y. , A. Lenz , and C. Koerner . 2014 The interaction between freezing tolerance and phenology in temperate deciduous trees. Front. Plant Sci. 5:doi:10.3389/fpls.2014.00541. 10.3389/fpls.2014.00541 PMC4192447 25346748

[ece32126-bib-0055] Way, D. A. , and R. A. Montgomery . 2015 Photoperiod constraints on tree phenology, performance and migration in a warming world. Plant, Cell Environ. 38:1725–1736. 25142260 10.1111/pce.12431

[ece32126-bib-0056] Wernberg, T. , B. D. Russell , P. J. Moore , et al. 2011 Impacts of climate change in a global hotspot for temperate marine biodiversity and ocean warming. J. Exp. Mar. Biol. Ecol. 400:7–16.

