# Supplementary material for: A phenological shift in the time of recruitment of the shipworm, Teredo navalis L., mirrors marine climate change
Source: Ecol Evol. 2016 May 10;6(12):3862–70. doi: 10.1002/ece3.2126 (PMC4972216; doi:10.1002/ece3.2126)
Supplement: Supplementary file 1 — Figure S1. Sea Surface Temperature (SST) in western Sweden for July–September 1970–2010. [file ECE3-6-3862-s001.docx]

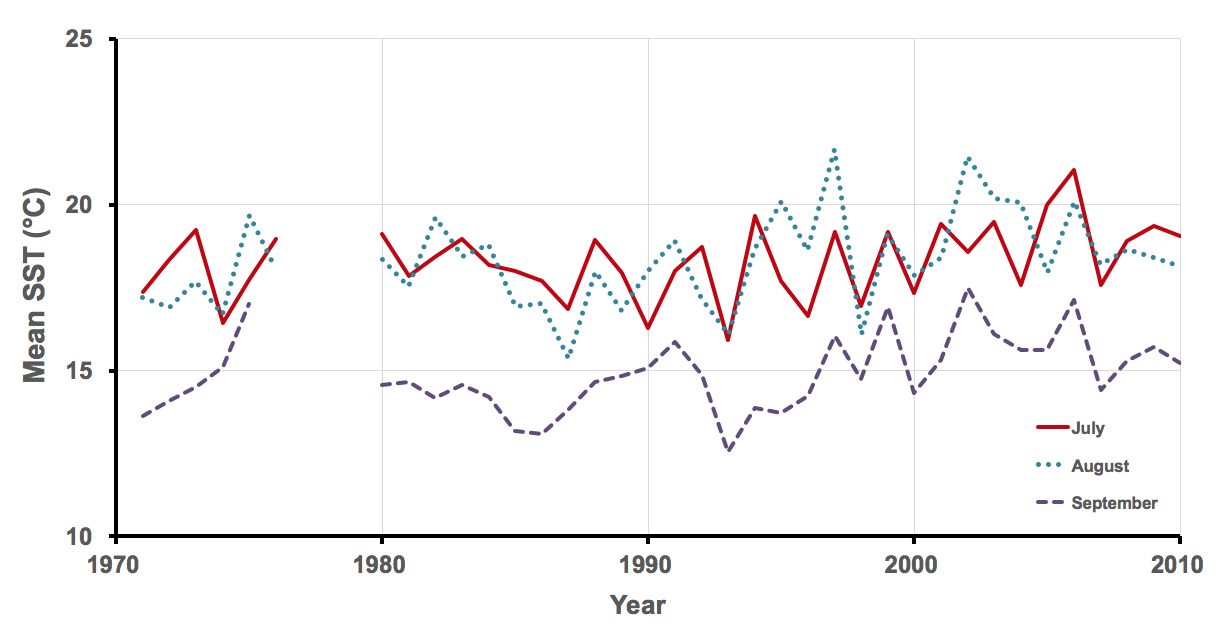


Figure S1 Sea Surface Temperature (SST) in western Sweden for Jul-Sept 1970-2010.

Means of daily hand-logged temperature data held at Sven Lovén Centre for Marine Sciences.
